# Synthesis of chromium-D-phenylalanine complex and exploring its effects on reproduction and development in *Drosophila melanogaster*

**DOI:** 10.3389/fphar.2023.1258081

**Published:** 2023-12-14

**Authors:** Shivsharan B. Dhadde, Mallinath S. Kalshetti, B. S. Thippeswamy

**Affiliations:** ^1^ Krishna Institute of Pharmacy, Krishna Vishwa Vidyapeeth (Deemed to be University), Karad, Maharashtra, India; ^2^ D.S.T.S. Mandal’s College of Pharmacy, Solapur, Maharashtra, India; ^3^ Department of Biomedical Science, College of Pharmacy, Shaqra University, Al-Dawadmi, Saudi Arabia

**Keywords:** trace element, chromium complex, dietary supplement, antioxidant, fruit flies

## Abstract

The present study was undertaken to explore the effect of Chromium-D-phenylalanine (Cr (D-phe)_3_) on the reproduction and development of *Drosophila melanogaster*. Cr (D-phe)_3_ was synthesized and characterized by infrared spectral analysis, melting point (DSC), and UV spectral analysis. *D. melanogaster* was raised in corn flour agar medium containing 0, 5, 10, 15, and 20 μg/mL of Cr (D-phe)_3_. The effect of Cr (D-phe)_3_ was evaluated by observing the larval period, pupal period, percentage of egg hatching, morphometric analysis of eggs, larvae, pupae and adults, fertility, fecundity, lifespan of the emerged flies, and levels of antioxidant enzymes such as catalase, glutathione-S-transferase (GST), and superoxide dismutase (SOD) in the supernatant of flies homogenate suspension. The study results indicate that Cr (D-phe)_3_ showed beneficial effects on reproduction and development in *D. melanogaster*. Cr (D-phe)_3_ significantly improved the larval period, pupal period, percentage of egg hatching, morphometric characters of the larva, pupa, and adult, fertility, fecundity, and lifespan of *D. melanogaster*. Moreover, Cr (D-phe)_3_ also significantly elevated the levels of catalase (*p* < 0.01), GST (*p* < 0.05), and SOD (*p* < 0.01) in *D. melanogaster*, and results were statistically significant at the dose of 15 μg/mL. The study results indicate that Cr (D-phe)_3_ has a positive effect on the reproduction and development of *D. melanogaster*. The literature review revealed that there is a strong relationship between the physiology of metabolism, oxidative stress and reproduction and development. Several studies propose that Cr(III) influences insulin sensitivity and thereby the metabolism of carbohydrates, proteins, and fats. Cr (D-phe)_3_ also has antioxidant and anti-inflammatory properties. Hence, the observed beneficial effects of Cr (D-phe)_3_ on reproduction and development of *D. melanogaster* may be attributed to its physiological effect on carbohydrate, protein, and lipid metabolism and its antioxidant and anti-inflammatory properties.

## 1 Introduction

Chromium (Cr) is one of the most common elements that occur in the earth’s crust and seawater. Cr exists in various oxidation states from 0 to VI; however, the most stable forms found in environmental and food samples are trivalent [Cr(III)] and hexavalent [Cr(VI)] (Peng and Yang, 2015).

Cr(VI) is highly toxic: it has an allergic, teratogenic, mutagenic, and carcinogenic impact on humans (Sun et al., 2015; Shams Ul Hassan et al., 2019). As per the recommendations of the International Agency for Research of Cancer (IARC) 2022, Cr(VI) is classified as Group 1—Carcinogenic to humans—and can cause lung cancer. Inhalation exposure to Cr(VI) can cause irritation, inflammation, and damage to the respiratory system. Prolonged skin contact can cause skin ulcers and allergic reactions (IARC 2022).

According to IARC-2022, Cr(III) is not carcinogenic to humans (IARC 2022) and is considered an essential trace element. Humans require about 30–40 µg of chromium per day for normal metabolism of glucose, fats, proteins, and nucleic acids (Peng and Yang, 2015; Nagarjun et al., 2017). Cr(III) encourages the action of insulin in tissues and also helps to maintain normal serum cholesterol levels (Peng and Yang, 2015; Nagarjun et al., 2017). Several studies propose that Cr(III) influences insulin sensitivity by enhancing insulin receptor binding and increasing both receptor numbers and intracellular insulin signalling. Additionally, it facilitates GLUT4 translocation through the AMPK-mediated pathway (Vincent, 2017; Lukaski, 2019). Cr(III) further contributes to elevated protein synthesis by activating post-receptor insulin signal transduction pathways and initiating phosphorylation cascades, thereby promoting anabolic protein responses in skeletal muscle (Lukaski, 2019). Furthermore, the supplementation of Cr(III) has been observed to impact lipid synthesis, leading to a reduction in the expression of genes linked to fatty acid synthesis in adipocytes. This effect extends to a decrease in fat accumulation in both the liver and skeletal muscle (Lukaski, 2019).

Cr demand drastically increases during long periods of stress such as pregnancy, infection, physical trauma, and strenuous exercise as additional Cr loss is found in these situations (Swaroop et al., 2019). Many people, such as athletes, diabetics, pregnant women, and the elderly, are especially at risk of Cr deficiency, leading to impaired insulin function, inhibition of protein synthesis and energy production, type 2 diabetes, and cardiovascular complications (Swaroop et al., 2019). Hence, supplementation of Cr through diet plays an important role in protecting against Cr deficiency-induced health complications in this group of people. In addition, Cr(III) compounds have demonstrated extensive applications in healthy blood sugar regulation, dietetics, and sports nutrition (Peng and Yang, 2015; Nagarjun et al., 2017; Swaroop et al., 2019).

Significant dietary sources of Cr (III) are present in a variety of foods, including whole-grain products, high-bran breakfast cereals, egg yolks, coffee, nuts, green beans, broccoli, meat, Brewer’s yeast, and certain brands of beer and wine. Cr(III) is also naturally occurring in many mineral or multivitamin supplements. As per the National Research Council (NRC), the Estimated Safe and Adequate Daily Dietary Intake (ESADDI) for Cr(III) ranges from 50 to 200 μg/day, corresponding to 0.83–3.33 μg/kg/day for adults weighing 60 kg. The Food and Drug Administration (FDA) has selected a Reference Daily Intake (RDI) of 120 μg/day for Cr (Swaroop et al., 2019).

Dietary Cr is not readily absorbed (about 0.5%–2%); the coupling of Cr to a suitable ligand increases its bioavailability (Peng and Yang, 2015; Swaroop et al., 2019). The pharmaceutical and dietary supplement market is growing fast by offering various chemical forms of Cr(III) compounds like Cr (III)-picolinate, Cr(III)-polynicotinate, Cr(III)-glycinate, Cr (III)-D-phenylalaninate, Cr (III)-histidinate, etc. (Maret, 2019). But Cr(III)-D-phenylalanine complex [Cr (D-phe)_3_] is reported as biologically safe compared to all others (Nagarjun et al., 2017).

Cr(III) compounds are commercially available either individually or as a component of herbal blends or mixed formulations with vitamins, minerals, or anabolic nutrients. In addition to traditional capsules or tablets, Cr(III) compounds are included in nutrition bars, chewing gum, and some sports drinks (Peng and Yang, 2015; Swaroop et al., 2019). Some scientific literature has raised concerns about the toxicities of these Cr (III) supplements, particularly regarding reproductive and developmental toxicities (Hepburn et al., 2003; Bailey et al., 2008).

Cr(III) complexes have the potential to affect insulin function, protein synthesis, and fat synthesis and reduce food intake (Vincent, 2017; Lukaski, 2019). These processes are often crucial in normal reproduction and fetal development (Sliwowska et al., 2014; Marshall et al., 2022). However, no studies have thoroughly evaluated and explored the effect of dietary supplement forms of Cr(III) in reproduction and development.

The *Drosophila melanogaster* (fruit fly) has long been a premier model for developmental biologists and geneticists (Rand et al., 2014). In addition, *D. melanogaster* is a model organism owing to its importance in toxicity studies: having a shorter life span and a large number of progenies is an ideal model for studying developmental and reproductive effects (Singh and Chowdhuri, 2017; Pb et al., 2020).

Hence, the present study was planned to synthesize Cr (D-phen)_3_ complex and explore its effects on reproduction and development in *D. melanogaster.*

## 2 Materials and methods

### 2.1 Chemicals

Chromium chloride (III) (Sd-fine-chem, Mumbai, India), D-phenylalanine, thiobarbituric acid, (Hi- Media Laboratories Pvt. Ltd, Mumbai, India), and 5,5-dithiobis-2- nitrobenzoic acid (Sigma-Aldrich, St. Louis, United States) were purchased. All other chemicals used were of analytical grade.

### 2.2 *Drosophila melanogaster*

Wild strains of *D. melanogaster* (W1118) were obtained from the National Centre for Biological Sciences (TIFR), Bangalore and sub-cultured into standard corn flour cream agar media and maintained at 25°C ± 1°C in a low-temperature incubator (REMI) at 65% humidity were used in the study.

### 2.3 Preparation of corn flour cream agar media

Corn flour (80 g), jaggery (70 g), agar (9 g), and yeast powder (15 g) were weighed separately and kept aside. Corn flour was added to a pressure cooker containing 1 L of warmed (∼35°C) distilled water and mixed thoroughly to avoid the formation of clumps. Subsequently, jaggery, agar, and yeast powder were also added, mixed thoroughly, and the pressure cooker was closed without a weight/whistle and kept on high heat for 5 min. Then cooker lid was opened carefully, the content was mixed thoroughly, and the lid was closed again and a whistle placed. Heating was continued for 25 min at low flame. The heat was turned off and it was left to cool for 15–20 min. The pressure in the cooker was released; the lid was opened and the cooked media was mixed thoroughly and cooled to 50°C–55°C. Then, the required amount of methyl para hydroxy benzoate (1.25 g) dissolved in a small quantity of ethanol (4 mL) and propionic acid (4.4 mL) were added to the media with thorough mixing. The prepared media was poured into glass vials (10 cm in length × 2 cm in diameter) to approximately 2 cm from the bottom.

### 2.4 Synthesis of Cr (D-phen)_3_ complex

Cr (D-phen)_3_ complex was systhesized as per the method prescribed in our previously published work (Nagarjun et al., 2017). Shortly, solutions of 2.6 g of CrCl_3_.6H2O in 50 mL water and 4.8 g, of D-phenylalanine in 50 mL water were mixed at 80°C and refluxed for 4 h. The homogeneous green reaction mixture was freeze–dried to obtain a greenish-violet solid and washed several times with acetone, dried in an air oven, and stored in a closed container for further use. The synthesized Cr (D-phen)_3_ complex was characterized by its infrared spectral analysis, melting point (differential scanning calorimeter), and UV spectral analysis [the results of characterization are given in our previous publication (Nagarjun et al., 2017)].

### 2.5 Exploring effects of Cr (D-phen)_3_ on reproduction and development

To evaluate the effect of Cr (D-phen)_3_ complex on reproduction and development, the flies of *D. melanogaster* (W1118) were raised in five replicates of standard corn flour cream agar medium containing 0, 5, 10, 15 and 20 μg/mL of Cr (D-phe)_3_ (equivalent weight of chromium) in glass vials (10 cm in length × 2 cm in diameter) and grouped as shown below.

Group I (normal control): flies were raised on a basal diet
Group II: flies were raised on a basal diet containing 5 μg/mL of Cr (D-phe)_3_
Group III: flies were raised on a basal diet containing 10 μg/mL of Cr (D-phe)_3_
Group IV: flies were raised on a basal diet containing 15 μg/mL of Cr (D-phe)_3_, and
Group V: flies were raised on a basal diet containing 20 μg/mL of Cr (D-phe)_3_

The effect of Cr (D-phe)_3_ was evaluated by determination of larval period, pupal period, percentage of egg hatching, morphometric analysis of egg, larvae, pupae, and adults, fertility, fecundity, lifespan, and levels of antioxidant enzymes such as catalase, glutathione-S-transferase, and superoxide dismutase.

#### 2.5.1 Determination of pupation and maturity percentage

Five replicates of standard corn flour cream agar medium containing different doses of Cr (D-phe)_3_ (0, 5, 10, 15, and 20 μg/mL) separately were prepared, and five pairs of 3-day-old flies were transferred to each vial. After permitting the flies to lay eggs for 8 h, they were allowed to exit the vials. The vials were then incubated at 25°C until the eggs hatched and larvae emerged, and they were allowed to grow until they reached the third instar. Larvae from each vial were collected carefully by washing with tap water through a fine-meshed muslin cloth and counted. The larvae were then moved to new media with the same concentrations and incubated once more until they pupated. The number of pupae appearing on the surface and next to the medium was counted. The vials were again incubated till flies were enclosed from the pupae. The number of adult flies emerged were counted by shaking them into flasks containing 70% ethanol. The percentage of emerging larvae, pupae, and adult flies was calculated. Additionally, the average time needed for the first larvae, pre-pupae, and adult to emerge from each concentration was noted, and the average duration of the larval period and pupal period were determined (Pb et al., 2020).

#### 2.5.2 Morphometric analysis of eggs

Five pairs of freshly enclosed flies that had spent their larval stage in media containing varying doses of Cr (D-phe)_3_ (0, 5, 10, 15, and 20 μg/mL) were moved to a new medium, where they were given 8 h to mate and lay their eggs. The adult flies were then permitted to leave the vials, and the length and width of the eggs were measured micrometrically (Pb et al., 2020).

#### 2.5.3 Percentage of hatched eggs

Newly enclosed flies who spent their larval period in respective media containing different doses of Cr (D-phe)_3_ (0, 5, 10, 15, and 20 μg/mL) were taken and five pairs of them were allowed to mate and lay eggs for 8 h. After allowing the adult flies to leave the bottles, the eggs laid were counted using a stereomicroscope. The bottles were then incubated at 25°C till the eggs hatched and the larvae reached the third instar stage. They were counted, the difference between the number of eggs laid and the number of larvae that emerged was determined, and then the percentage of eggs that hatched was computed using the pooled data from the five replicates (Pb et al., 2020).

#### 2.5.4 Morphometric variation in larvae, pupae and adults

Similar media with different Cr (D-phe)3 dosages were made in flat-bottomed flasks with a 100 mL capacity. Each container contained 20 adult fly pairs that were allowed to mate and lay eggs for 8 h. The bottles were incubated until the larvae emerged after the flies were permitted to leave them. After the first moulting, seven larvae were taken from each bottle and measured micrometrically for length and width. Sampling was repeated every 12 h for eight more times after which they pupate. The length and breadth of the pupae were similarly measured and few pupae were allowed to eclose to become sample adults to measure their chest width after 3 days (Pb et al., 2020).

#### 2.5.5 Determination of fitness

Five replicates of standard corn flour cream agar medium containing four doses of Cr (D-phe)_3_ (0, 5, 10, 15, and 20 μg/mL) separately were prepared. Five pairs of 3-day-old flies were transferred to each vial. The flies were allowed to exit the vials after allowing them to lay eggs for 8 h. Eggs from each vial were collected carefully by washing with tap water through a fine-meshed muslin cloth (Pb et al., 2020).

An equal number of eggs were then transferred to fresh media containing the same concentrations and again incubated at 25°C. Three components were measured to assess fitness: dynamics of eclosion, developmental time, and egg-to-adult survival. Egg-to-adult survival is expressed as the ratio of adult flies to the number of eggs placed in each bottle (Pb et al., 2020).

#### 2.5.6 Determination of fertility and fecundity

The flies were allowed to grow in their respective medium for five generations. The fifth-generation adults were collected, and five pairs of male and female flies were transferred to vials containing fresh corn cream agar medium in five replicates. The male flies after 48 h of mating were allowed to exit, and the female flies were transferred to fresh vials every 24 h the average number of eggs laid per female per day was calculated to determine the fecundity. The vials were incubated at 25°C till the larvae were pupated and eclosed to emerge as adult flies. The number of flies was quantified by capturing them in 70% ethanol. Fertility was determined as the average number of adult flies that emerged from each pair of flies (Pb et al., 2020).

#### 2.5.7 Determination of lifespan

For assessing lifespan, standard corn flour cream agar medium containing Cr (D-phe)_3_ (0, 5, 10, 15, and 20 μg/mL) was prepared, and 3-day-old flies were transferred to each vial with five replicates. All the vials were maintained at 25°C, and every second day the flies were transferred to new vials containing fresh media. Mortality per day was noted in each vial, and survival time for each group was calculated (Pb et al., 2020).

#### 2.5.8 Determination of antioxidant enzymes

Assays of antioxidant enzymes were performed in the homogenate of 20 flies in 500 µL of Tris buffer (20 mM, pH 7.0) and subsequent centrifugation at 20,000 g for 5 min at 4°C. This supernatant was used to measure levels of catalase (CAT) based on its ability to catalyze the breakdown of hydrogen peroxide (H_2_O_2_) into water (H_2_O) and molecular oxygen (O_2_) (Veerapur et al., 2011; Nagakannan et al., 2012; Shivasharan et al., 2013). Glutathione S-transferase (GST) activity was measured as described in the published protocol and the results were expressed as nmol of CDNB conjugate formed/min/mg protein (Shivasharan et al., 2013). Superoxide dismutase (SOD) activity was estimated by measuring its ability to catalyze the dismutation of superoxide radicals (O_2_−​) into oxygen (O_2_​) and hydrogen peroxide (H_2_O_2_)(Nagakannan et al., 2012). Total protein content was assessed following the Bradford method using bovine serum albumin as standard (Dhadde et al., 2016).

### 2.6 Statistical analysis

The values were expressed as mean ± SEM. The statistical analysis was carried out by One-way analysis of variance (ANOVA) followed by Tukey’s *post hoc* test. *p* < 0.05 was considered statistically significant.

## 3 Results

### 3.1 Pupation and maturity percentage

#### 3.1.1 Larval period

**FIGURE 1:**
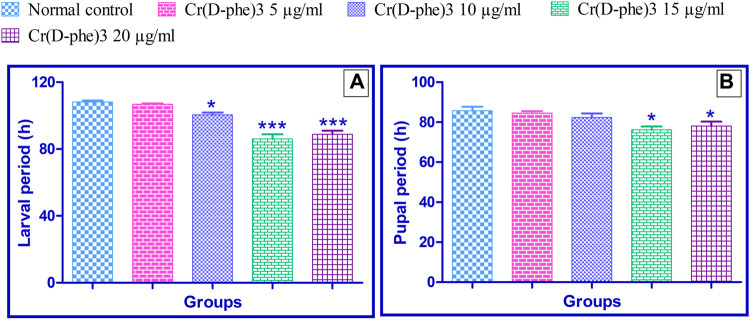
Effect of Cr (D-phe)_3_ supplementation on the **(A)** larval period and **(B)** pupal period of *D. melanogaster.* Values are expressed in mean ± SEM, *p < 0.05 and ***p < 0.001 when compared with normal control group. One-way analysis of variance (ANOVA) followed by Tukeys’ *post hoc* test.

The normal control flies spent an average of 108.2 ± 0.86 h in the larval period. This time was reduced by 1%–20% in the files supplemented with Cr (D-phe)_3._ The flies supplemented with 10, 15, and 20 μg/mL of Cr (D-phe)_3_ showed significantly (*p* < 0.05, *p* < 0.001, and *p* < 0.001) reduced larval periods compared with the normal control. The results of the effect of Cr (D-phe)_3_ on flies in the larval period are shown in Figure 1A.

#### 3.1.2 Pupal period

The normal control group spent an average of 85.80 ± 2.01 h in the pupal period. This time was reduced by 1.39%–11.18% in the group supplemented with different concentrations of Cr (D-phe)_3._ The flies supplemented with 15 and 20 μg/mL of Cr (D-phe)_3_ showed significantly (*p* < 0.05 and *p* < 0.05) reduced pupal periods compared with the normal control. The results of the effect of Cr (D-phe)_3_ on the flies’ pupal period duration are shown in Figure 1B.

#### 3.1.3 Percentage of larvae emerged

**FIGURE 2:**
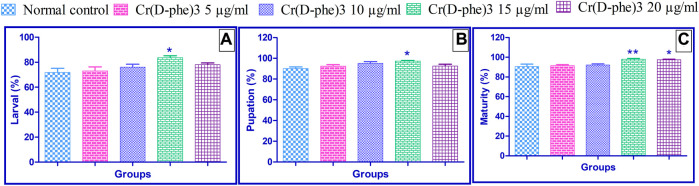
Effect of Cr (D-phe)_3_ supplementation on the **(A)** percentage of larvae emerging; **(B)** percentage growth of pupae from larvae; **(C)** percentage growth of adult flies from pupae of *D. melanogaster.* Values are expressed in mean ± SEM, *p < 0.05 and **p < 0.01 when compared with normal control group. One-way analysis of variance (ANOVA) followed by Tukeys’ *post hoc* test.

In the normal control group, 71.80% ± 3.35% of laid eggs emerged as larvae. Supplementation of Cr (D-phe)_3_ improved the emergence of larvae from the laid eggs. The percentage of larvae at 5 μg/mL was 73% ± 3.20%, at 10 μg/mL was 76% ± 2.34%, at 15 μg/mL was 83.60% ± 1.50% (*p* < 0.05), and at 20 μg/mL was 78.0% ± 1.41%. The results of the effect of Cr (D-phe)3 on a percentage of larvae emerging are shown in Figure 2A.

#### 3.1.4 Percentage of pupae emerged

In the normal control group, 90.20% ± 1.46% of larvae were grown to pupae. Supplementation of Cr (D-phe)_3_ improved the emergence of pupae from the larvae. The percentage of growth of pupae from larvae in 5 μg/mL was 92.40% ± 1.40%, in 10 μg/mL was 95.20% ± 1.65%, in 15 μg/mL was 97.40.60% ± 1.50% (*p* < 0.05), and in 20 μg/mL was 92.60% ± 1.69%. The results of the effect of Cr (D-phe)_3_ on percentage growth of pupae from larvae are shown in Figure 2B.

#### 3.1.5 Percentage of adult flies emerged

In the normal control group, 90.60% ± 2.37% of pupae matured and became adult flies. Supplementation of Cr (D-phe)_3_ improved the emergence of adult flies from the pupae. The percentage of growth of adult flies from pupae in 5 μg/mL was 91.40% ± 1.03%, in 10 μg/mL was 92.20% ± 1.06%, in 15 μg/mL was 98.0% ± 0.07% (*p* < 0.01), and in 20 μg/mL was 97.40% ± 0.67% (*p* < 0.05). The results of the effect of Cr (D-phe)_3_ on percentage growth of adult flies from pupae are shown in Figure 2C.

### 3.2 Morphometric analysis of eggs

**FIGURE 3:**
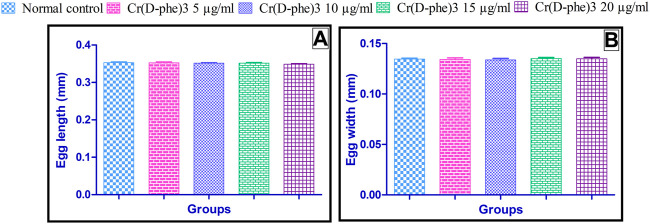
Effect of Cr (D-phe)_3_ supplementation on morphometric analysis of eggs of *D. melanogaster,* **(A)** Egg length and **(B)** egg width. Values are expressed in mean ± SEM, statistically non-significant when compared with normal control group. One-way analysis of variance (ANOVA) followed by Tukeys’ *post hoc* test.

The results of morphometric analysis of egg revealed that there is no statistically significant change in the length and width of the egg observed in Cr (D-phe)_3_ supplemented flies compared to the normal control group. The results of the effect of Cr (D-phe)_3_ on the morphometric analysis of eggs are shown in Figure 3.

### 3.3 Percentage of hatched eggs

**FIGURE 4:**
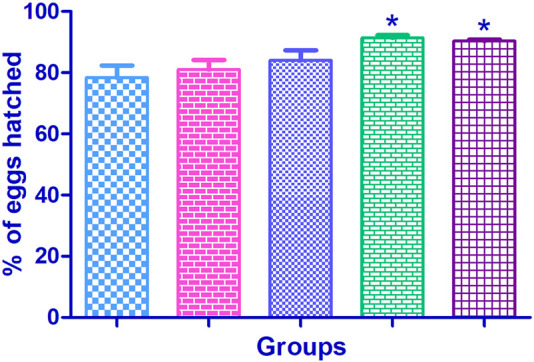
Effect of Cr (D-phe)_3_ supplementation on the percentage of egg hatching of *D. melanogaster.* Values are expressed in mean ± SEM, *p < 0.05 when compared with normal control group. One-way analysis of variance (ANOVA) followed by Tukeys’ *post hoc* test.

In the normal control group, the percentage of egg hatching was found to be 78.40% ± 3.94%. The egg hatching was improved in Cr (D-phe)_3_ supplemented group compared to the normal control. The egg hatching was found to be 81.0% ± 3.17% at 5 μg/mL, 84.0% ± 3.39% at 10 μg/mL, 91.40% ± 0.92% (*p* < 0.05) at 15 μg/mL, and 90.40% ± 0.50% (*p* < 0.05) at 20 μg/mL. The results of the effect of Cr (D-phe)_3_ on the percentage of egg hatching are shown in Figure 4.

### 3.4 Morphometric variation in larvae, pupae and adults

The results of morphometric variation in larvae, pupae, and adults revealed that there is no statistically significant variation in the length and width of larvae and pupae and chest width of adults supplemented with Cr (D-phe)_3_ compared to the normal control group. The results of the effect of Cr (D-phe)_3_ on the morphometric variation in larvae, pupae, and adults are shown in Table 1.

**TABLE 1:** Morphometric variation in larvae, pupae, and adults.

Group	Larvae		Pupae		Adult flies chest width
	Length (mm)	Width (mm)	Length (mm)	Width (mm)	
Normal control	4.33 ± 0.13	1.74 ± 0.01	3.66 ± 0.07	1.14 ± 0.05	1.07 ± 0.08
Cr (D-phe)_3_ 5 μg/mL	4.32 ± 0.13	1.73 ± 0.01	3.62 ± 0.05	1.18 ± 0.04	1.08 ± 0.04
Cr (D-phe)_3_ 10 μg/mL	4.49 ± 0.12	1.74 ± 0.01	3.75 ± 0.05	1.18 ± 0.05	1.07 ± 0.05
Cr (D-phe)_3_ 15 μg/mL	4.64 ± 0.13	1.74 ± 0.01	3.76 ± 0.07	1.23 ± 0.04	1.10 ± 0.05
Cr (D-phe)_3_ 20 μg/mL	4.51 ± 0.10	1.72 ± 0.01	3.68 ± 0.06	1.20 ± 0.04	1.07 ± 0.05

Values are expressed in mean ± SEM, statistically non-significant when compared with normal control group. One-way analysis of variance (ANOVA) followed by Tukeys’ *post hoc* test.

### 3.5 Fitness

**FIGURE 5:**
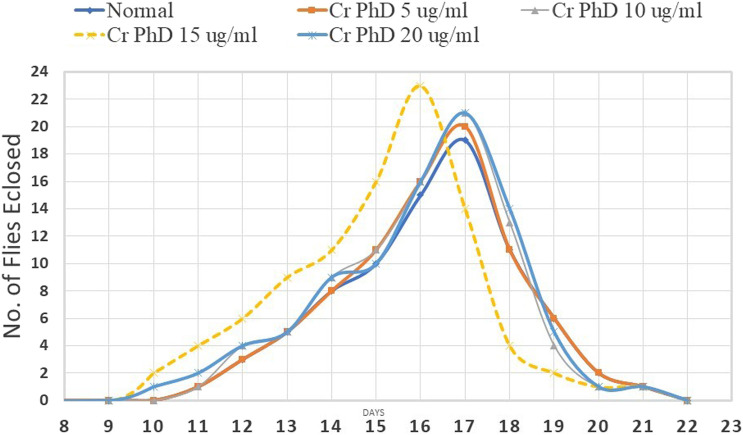
Effect of Cr (D-phe)_3_ supplementation on dynamics of eclosion of *D. melanogaster.* Values are expressed in mean ± SEM, *p < 0.05 when compared with normal control group. One-way analysis of variance (ANOVA) followed by Tukeys’ *post hoc* test.

Flies emerged from day 10 to day 21 in all groupsexcept for the Cr (D-phe)_3_ 15 μg/mL supplemented group; in all other groups, the maximum number of flies emerged on day 17, in the Cr (D-phe)_3_ 15 μg/mL supplemented group the highest number of flies emerged on day 16. The effect of Cr (D-phe)_3_ on the dynamics of eclosion of flies is shown in Figure 5.

**FIGURE 6:**
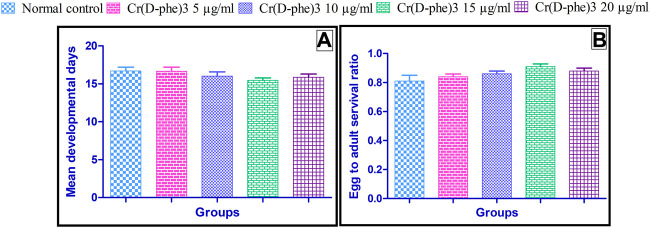
Effect of Cr (D-phe)_3_ supplementation on **(A)** mean developmental time and **(B)** ratio of adult flies emerged to the number of eggs of *D. melanogaster.* Values are expressed in mean ± SEM, statistically non-significant when compared with normal control group. One-way analysis of variance (ANOVA) followed by Tukeys’ *post hoc* test.

The mean developmental time required for normal control flies was 16.7 ± 0.49 days. There was no statistically significant change in the mean developmental time observed in the flies supplemented with different doses of Cr (D-phe)_3_; however, there was a slight decrease in the mean developmental time required in treated groups. The effect of Cr (D-phe)_3_ mean developmental time of flies is shown in Figure 6A.

The ratio of adult flies to the number of eggs was found to be 0.81 ± 0.04 in the normal control. This ratio was slightly increased in the flies supplemented with Cr (D-phe)_3_ and it was found to be 0.84 ± 0.02 at 5 μg/mL, 0.86 ± 0.02 at 10 μg/mL, 0.91 ± 0.02 at 15 μg/mL, and 0.88 ± 0.02 at 20 μg/mL. The change in ratio of adult flies to the number of eggs was not statistically significant when compared to the normal control. The results of effect of Cr (D-phe)_3_ on the ratio of adult flies to the number of eggs is shown in Figure 6B.

### 3.6 Fertility and fecundity

**FIGURE 7:**
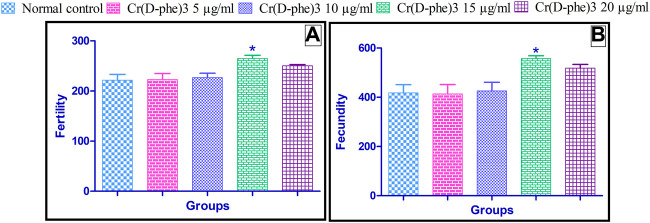
Effect of Cr (D-phe)_3_ supplementation on **(A)** fertility and **(B)** fecundity of *D. melanogaster*. Values are expressed in mean ± SEM, *p < 0.05 when compared with normal control group. One-way analysis of variance (ANOVA) followed by Tukeys’ *post hoc* test.

The fecundity parameter is calculated as the number of offspring that can be produced by a single-mated female fly. The egg-producing capability was considerably improved to a great extent in flies supplemented with Cr (D-phe)_3_ when compared with normal control flies. Similarly, the fertility parameter is expressed as the number of offspring that have developed from a single fly pair. Fertility was significantly increased in flies supplemented with 15 μg/mL of Cr (D-phe)_3_ when compared with normal control flies. The effect of Cr (D-phe)_3_ on the fertility and fecundity of flies is shown in Figure 7.

### 3.7 Lifespan

**FIGURE 8:**
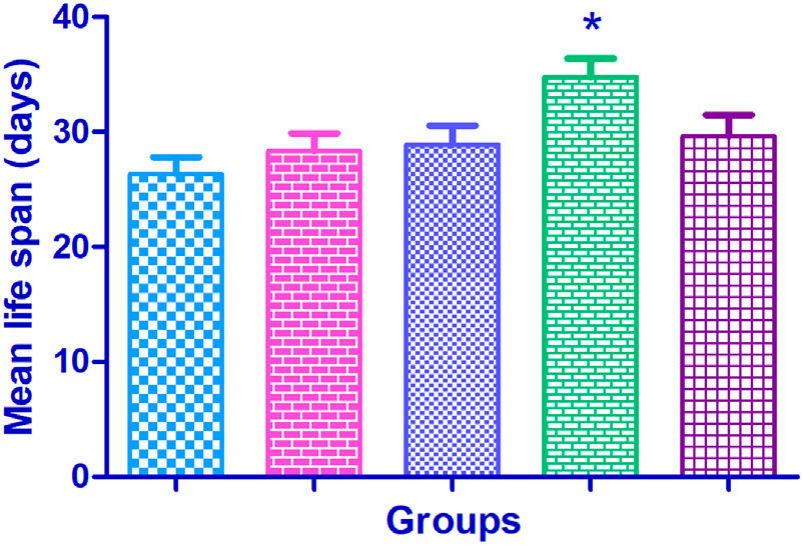
Effect of Cr (D-phe)_3_ supplementation on mean life span of *D. melanogaster*. Values are expressed in mean ± SEM, *p < 0.05 when compared with normal control group. One-way analysis of variance (ANOVA) followed by Tukeys’ *post hoc* test.

**FIGURE 9:**
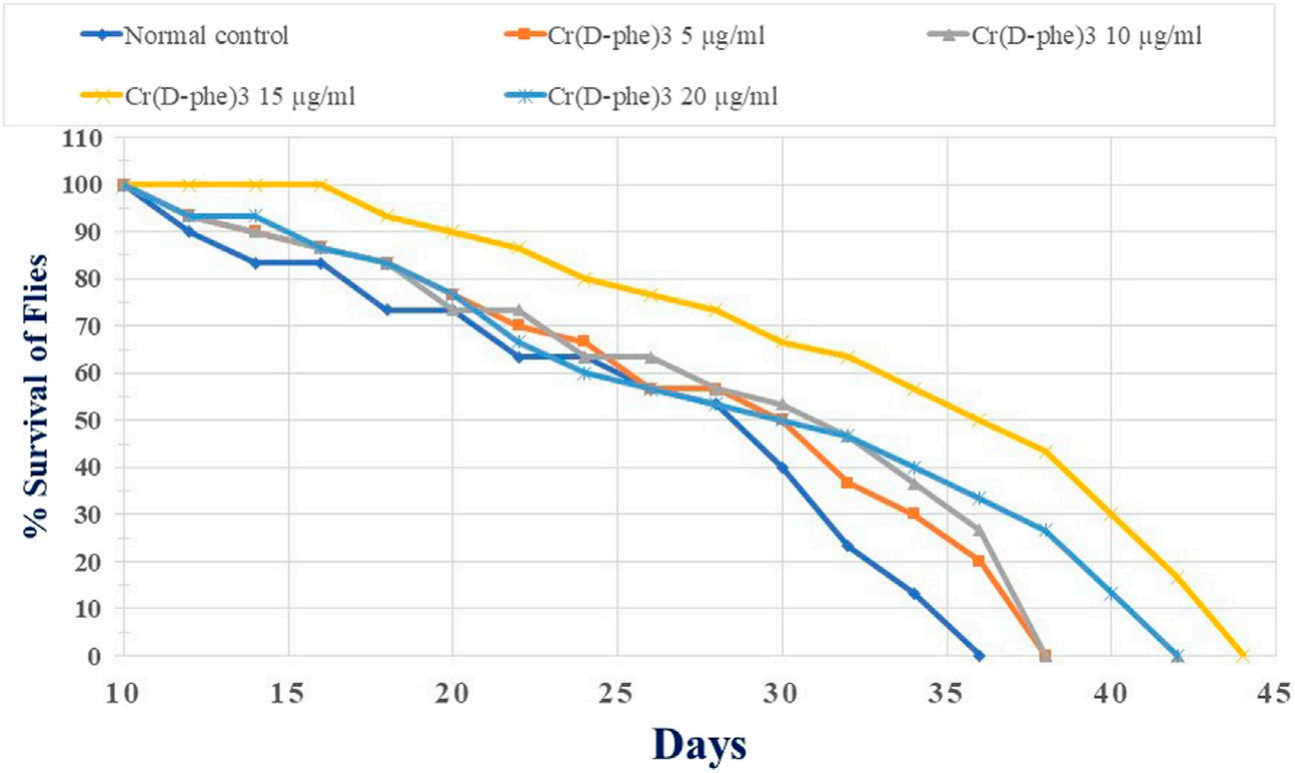
Effect of Cr (D-phe)_3_ supplementation on % survival of flies with increasing days of *D. melanogaster*. Values are expressed in mean ± SEM.

The mean life span in the normal control flies was found to be 47.06 ± 1.49 days. Supplementation with Cr (D-phe)_3_ increased the life span in the flies by 4.22%–16.47%; it was found to be 49.06 ± 1.54 days at 5 μg/mL, 50.19 ± 1.69 days at 10 μg/mL, 54.81 ± 1.82 (*p* < 0.05) days at 15 μg/mL, and 50.59 ± 1.94 days at 20 μg/mL. The results of effect of Cr (D-phe)_3_ on mean life span of flies are shown in Figure 8, and the % survival of flies with increasing days is shown in Figure 9.

### 3.8 Antioxidant enzymes

**FIGURE 10:**
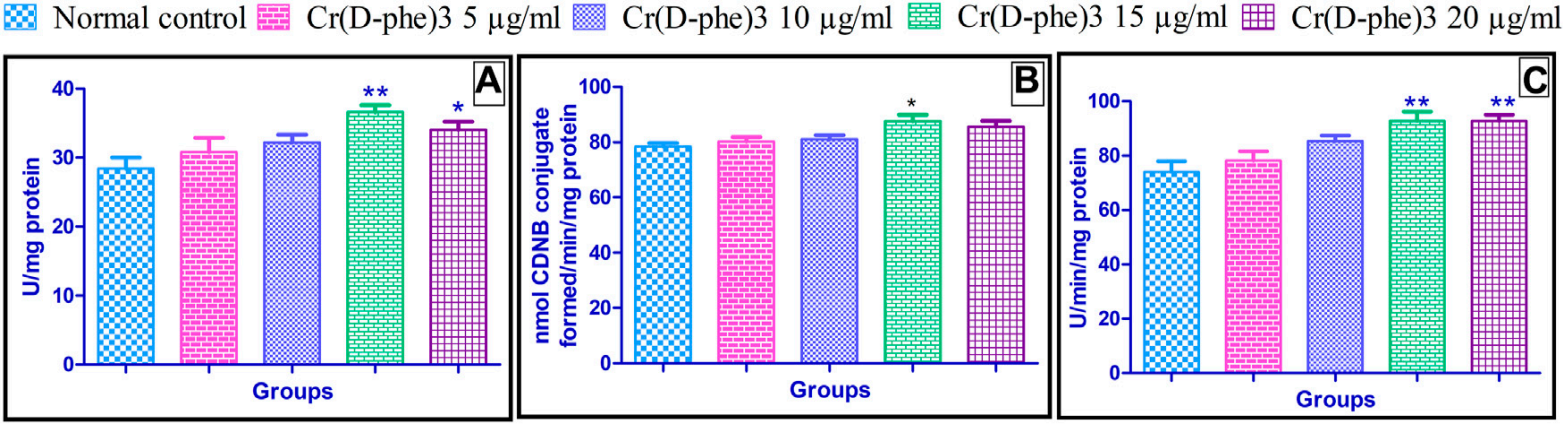
Effect of Cr (D-phe)_3_ supplementation on antioxidant levels **(A)** catalase; **(B)** glutathione-S-transferase; **(C)** superoxide dismutase in *D. melanogaster.* Values are expressed in mean ± SEM, *p < 0.05 and **p < 0.01 when compared with normal control group. One-way analysis of variance (ANOVA) followed by Tukeys’ *post hoc* test.

Antioxidant enzymes such as catalase, GST, and SOD levels were increased in the flies supplemented with different doses of Cr (D-phe)_3_. The increase in the levels of antioxidant enzymes was in the range of 8.45%–28.87% (catalase), 2.29%–11.73% (GST), and 5.67%–25.40% (SOD). Supplementation of Cr (D-phe)_3_ at the dose of 15 μg/mL showed significantly improved levels of catalase (*p* < 0.01), GST (*p* < 0.05), and SOD (*p* < 0.01). Cr (D-phe)_3_ at the dose of 15 mg/kg showed statistically significant improvement in terms of the levels of catalase (*p* < 0.05) and SOD (*p* < 0.01). The effect of Cr (D-phe)_3_ supplementation on antioxidant levels is shown in Figure 10.

## 4 Discussion

Chromium (III) is recognised as a unique micronutrient for its advantageous role in human nutrition, acting as an essential cofactor in the action of insulin as well as providing nutritional support for energy, glucose, and lipid metabolism (Piotrowska et al., 2018). Currently, chromium (III) salts, including chelates, have shown a wide range of medical applications, particularly in the areas of dietetics, sports nutrition, and good blood sugar regulation (Swaroop et al., 2019).

Over the last 2 decades, Cr nutritional supplements become very popular for weight loss, muscle development and diabetes (Peng and Yang, 2015; Swaroop et al., 2019). Various chemical forms of Cr(III), including Cr picolinate, Cr histidinate, Cr nicotinate, Cr glycinate and Cr - D-phenylalanine have been used worldwide as Cr supplement (Peng and Yang, 2015; Swaroop et al., 2019). As mentioned above, Cr (D-phe)_3_ is biologically safe compared to other Cr-complexs.

In the present study, Cr (D-phe)_3_ was evaluated for its effects on reproduction and development in *D. melanogaster.* The study results suggest that Cr (D-phe)_3_ supplementation at the dose of 5, 10, 15, and 20 μg/mL did not show any harmful effects on the reproduction and development of *D. melanogaster.* Instead, the supplementation of Cr (D-phe)_3_ showed positive effects on the reproduction and development of *D. melanogaster.* This was evidenced by its positive effects on pupation and maturity percentage, percentage of pupae and adult flies that emerged, percentage of eggs hatched, and antioxidant enzyme levels in the Cr (D-phe)_3_ supplemented flies compared to the normal control.

The metabolism of carbohydrates, proteins, and fats plays a crucial role in providing the energy required for reproductive processes and supporting the development of a healthy foetus (Sliwowska et al., 2014; Marshall et al., 2022). Maintaining balanced blood sugar levels, insulin sensitivity, and overall metabolic health is important for both male and female reproductive function. Protein metabolism is necessary for the synthesis and regulation of reproductive hormones, which orchestrate the menstrual cycle, ovulation, and support pregnancy (Sliwowska et al., 2014). Additionally, protein metabolism is essential for maintaining the structural integrity of maternal tissues during pregnancy (Sliwowska et al., 2014).

Oxidative stress plays an important role in male and female subfertility (Tremellen, 2008; Poston et al., 2011; Smits et al., 2018). Reduced fertility in men has been associated with oxidative damage to sperm (Poston et al., 2011). Excessive ROS synthesis will impair oocyte maturation (meiosis II), and an inadequate intracellular antioxidant capacity can limit successful ovulation and fertilization (Poston et al., 2011). Reduced levels of endogenous antioxidants such as SOD, GSH, and Glutathione peroxidase (GPX) in sperm and seminal plasma were found in infertile males (Tremellen, 2008). Cr-Dphe has very good antioxidant and anti-inflammatory properties (Nagarjun et al., 2017). In the present study, supplementation of Cr (D-phe)_3_ to *D. melanogaster* improved the levels of endogenous antioxidants well supports the above findings.

Phenylalanine is an essential nutrient and is an important precursor to many biomolecules such as neurotransmitters, hormones, and enzymes, and is also a precursor for many vitamins and minerals (Litwack, 2018). In addition, it has been shown to have a positive effect on the metabolism of carbohydrates, proteins, and fats. Research has found D-Phe has anti-inflammatory, anti-oxidant, and neuroprotective effects. It also has a positive effect on the immune, cardiovascular, and nervous systems (Litwack, 2018). Moreover, phenylalanine is important for the synthesis of thyroid hormones that control metabolic processes, thereby influencing the growth of different body structures; feed efficiency; oxygen consumption; synthesis and metabolism of proteins, carbohydrates and lipids; thermogenesis; and acclimation to environmental changes (Nagarjun et al., 2017; Litwack, 2018). All these effects of D-Phe also contributed to the beneficial effects observed in the present study.

Among the different tested doses of Cr (D-phe)_3_, a dose of 15 μg/mL showed optimum results based on the evaluated parameters. The beneficial effect of Cr (D-phe)_3_ increases with its increasing dose from 5 to 15 μg/mL and a further increase in the dose to 20 μg/mL did not show an increase in response; instead, 20 μg/mL slightly reduced the beneficial actions compared to the 15 μg/mL. This indicates that up to a certain dose, chromium shows a beneficial effect that the action decreases with a further increase in dose.

## 5 Limitation of the present study

This study was conducted on flies, hence the effect of Cr (III) supplements in higher animals needs to be conducted for further confirmation of the study outcome. Moreover, in this study, only one complex of chromium was evaluated. The comparison of the effect of different supplementary forms of chromium compound needs to be explored.

## 6 Conclusion

In conclusion, the present study data indicated that supplementation of Cr (D-phe)_3_ to *D. melanogaster* did not show any reproductive and developmental toxicities. Instead, the Cr (D-phe)_3_ showed beneficial effects in the reproduction and development of *D. melanogaster.* The literature review revealed that there is a strong relationship between the physiology of metabolism and oxidative stress. Several studies propose that Cr(III) influences insulin sensitivity and thereby metabolism of carbohydrates, proteins, and fats and also has antioxidant and anti-inflammatory properties. Hence, the observed beneficial effects of Cr (D-phe)_3_ on reproduction and development of *D. melanogaster* may be attributed to its physiological effect on carbohydrate, protein, and lipid metabolism and its antioxidant and anti-inflammatory properties. The effect of Cr (III) supplements in the higher animals needs to be conducted for further confirmation of the study outcomes.

## Data Availability

The original contributions presented in the study are included in the article/Supplementary material, further inquiries can be directed to the corresponding authors.
